# Carbon dots as fluorescent nanoprobes for assay of some non-fluorophoric nitrogenous compounds of high pharmaceutical interest

**DOI:** 10.1186/s43088-023-00346-z

**Published:** 2023-01-17

**Authors:** Rana M. Moustafa, Wael Talaat, Rasha M. Youssef, Miranda F. Kamal

**Affiliations:** 1 Department of Pharmaceutical Chemistry, Faculty of Pharmacy and Drug Manufacturing, Pharos University in Alexandria, Alexandria, Egypt; 2 Department of Pharmaceutical Analytical Chemistry, Faculty of Pharmacy, Damanhour University, Damanhour, Egypt; 3 Department of Pharmaceutical Analytical Chemistry, Faculty of Pharmacy, Alexandria University, Alexandria, Egypt

**Keywords:** Azithromycin, Rasagiline mesilate, Luminescent probe, Carbon nanodots, Quenching

## Abstract

**Background:**

Carbon dots, CDs, have excellent photoluminescence properties, good biocompatibility, low toxicity and good light stability. The optical, magnetic and electronic properties of CDs make them a hugely relevant tool to be used in pharmaceutical analysis, bioimaging, drug delivery, and other fields. The fluorescence of carbon nanodots makes it suitable for assay of some nitrogenous compounds of high pharmaceutical interest. In this work, we develop simple, fast and green spectrophotometric methods for quantification of Azithromycin and Rasagiline mesilate using synthesized fluorescent CDs from garlic peels.

**Results:**

The spectrometric methods depend on stoichiometric reactions of both drugs with fluorescent CDs. Carbon dots exhibit a declared absorption peak *λ*max at 238 nm and potent fluorimetric emission at *λ*em 528 nm, upon excitation at *λ*ex 376 nm. Drugs’ concentrations in ppm are efficiently calculated using Stern–Volmer Equation. Decrease in fluorescence (Δ*F* = *F*_o_ − *F*) and the *F*-ratio values are linearly correlated to molar concentration of each quencher (drug). A significant linear diminish in the dots’ measured absorbance and fluorimetric emission values was observed. Validation of all the developed methods was according to the ICH guidelines.

**Conclusions:**

In a new way, this work successfully indicates, spectrometric methods for rapid detection of two non-fluorophoric nitrogenous compounds using potent carbon nanodots. Consequently, these green developed methods offer several benefits as simplicity, ease of quantification, accuracy and precision that encourage the application of the developed methods in routine analysis of Azithromycin and Rasagiline mesilate in quality control laboratories as analytical tool.

**Supplementary Information:**

The online version contains supplementary material available at 10.1186/s43088-023-00346-z.

## Background

CDs are non-dimensional nanoparticles with a size of less than 10 nm. Due to their applications in computer science and electronics, fluorescent carbon nano dots have sparked a lot of interest in the last decade [1, 2]. Compared to other fluorogenic probes, CDs step forward by their inherent features; chemical stability, inertness, significant luminescent emissions, least toxicity, ease of synthesis, high biocompatibility and photostability [3–7]. Furthermore, they possess free aqueous solubility as a result of hanging hydroxyl and carboxylic groups at the surface, high safe carbon content (99.9%) relative to inorganic metal nanoparticles and finally the affordability endears the CDs’ participation in nowadays targeted green analytical methodologies [8].

Generally, there are many methods for synthesizing CDs, including electrochemical, chemical oxidation, laser ablation, and arc discharge. Furthermore, when seeking a synthesis approach to obtain CDs, a number of criteria need to be taken into account. The possibility of carbonaceous aggregation, which is frequently formed during the carbonization process, emerges in the synthesis of CDs. Synthesis techniques including the hydrothermal route, organic pyrolysis, and microwave assisted approach can solve this problem. These methods have the ability to regulate the homogeneity and size of CDs in solvents [9].

Nowadays, scientific community’s interest has been directed towards synthesis, direct and indirect CDs’ assays. The present labour targets the insightful use of CDs for optic nano sensing of two non-fluorophoric nitrogenous compounds of high pharmaceutical interest; Azithromycin (AZN) and Rasagiline mesilate (RSGL) in both pure bulk and medical products. Quantitative CDs quenching is related to the added traces of each drug in stoichiometric reactions.

This article enfolds the direct use of green synthesized CDs [10] as spectrophotometric and fluorescent probes for both drugs’ analysis. The used nano CDs were already synthesized from garlic peels, as natural precursor, as described by Gaber et al. [11]. Detailed steps of synthesis of the used nanodots are provided in Additional file 1.

**Fig. 1:**
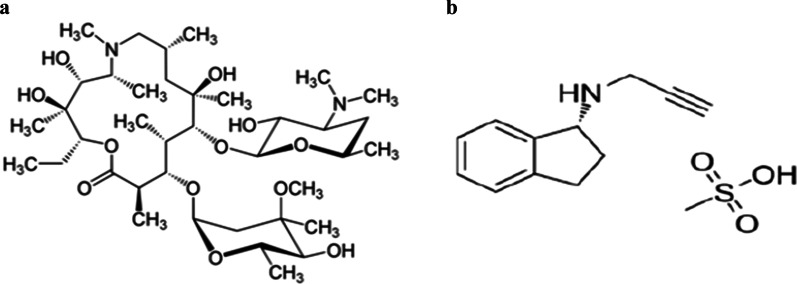
**a** Chemical structures of Azithromycin and **b** Rasagiline mesilate

AZN belongs to the macrolide family of antibiotics. It is mainly indicated for respiratory, enteric and genitourinary infections [12, 13]. AZN has been included in the solidarity concomitant Covid-19 protocol [14] since March, 2020. AZN, chemically named C_38_H_72_N_2_O_12_, Mwt 748.98 g/mol. Its IUPAC name is (2R, 3S, 4R, 5R, 8R, 10R, 11R, 12S, 13S, 14R)-13-[(2,6-Dideoxy-3-C-methyl-3-*O*-methyl-α-l-ribo-hexopyranosyl)oxy]-2-ethyl-3,4,10-trihydroxy-3,5,6,8,10,12,14-heptamethyl-11-[[3,4,6-trideoxy-3-(dimethylamino)-β-d-xylo-hexopyranosyl]oxy]-1-oxa-6 azacyclopentadecan-15-one; and Fig. 1 depicts its chemical structure. AZN lacks chromophore for direct UV absorption. So, its quantification represents a real analytical challenge.

RSGL is a novel molecule that works as an irreversible monoamine oxidase inhibitor to treat idiopathic Parkinson's disease [15–17]. Chemical name for RSGL is (1*R*)-*N*-(prop-2-yn-1-yl)-2,3-dihydro-1*H*-inden-1-amine; methane sulfonic acid, (Fig. 1). Literature reveals few spectrophotometric methods [18–23] and two fluorimetric assays [24, 25] for determination of AZN in bulk and formulations. A detailed review for RSGL estimation [26] is reported, without direct fluorimetric technique. RSGL has been determined by LC-fluorimetric detection in rat plasma for pharmacokinetic studies [27]. This endears developing novel selective, sensitive, methods of AZN and RSGL determination in routine quality control. Herein, CDs act as potent spectrometric sensors for non-fluorophoric nitrogenous compounds. Both drugs react stoichoimetrically with CDs, resulting in a linear diminish in the dots measured absorbance and fluorimetric emission values. The present study achieves green and economic direct AZN and RSGL optical sensing in both pure and pharmaceutical dosage forms.

## Methods

### Instrumentations and materials

On a Shimadzu UV Spectrophotometer (UV-1800) with 1.0 cm quartz cuvettes, all spectral measurements were taken. Electronic Axis analytical balance was employed for weighing the materials. Cary Eclipse Fluorescence Spectrophotometer manufactured by Agilent technology containing a Xenon flash lamp, lamp pulse width at half peak height ~ 2 µs, peak power equal to 75 kW and Czerny–Turner monochromators with 12.5 cm focal width. Separate monochromators for excitation and emission and Secondary light is eliminated by constructed excitation and emission filters. Cary Eclipse Software was used for recording spectra.

AZN and RSGL pure standards were purchased from Sigma Aldrich (St. Louis, MO, USA). HPLC grade Methanol (Merck, Darmstadt, Germany) was used. Deionized water was obtained from Science Park Unit, Faculty of Pharmacy, Alexandria University. All solutions were prepared in deionized water.

### Preparation and characterization of CDs

Synthesis and specific characterization of used CDs are clarified in Additional file 1.

### Preparation of standard stock solution of CDs

Standard stock solution of synthesized CDs solid powder 50 µg/mL was made with deionized water. A freshly-prepared stock solution of CDs was made daily before labour.

### Preparation of standard stock solutions of AZN and RSGL (100 mg%)

Standard stock solutions of each drug; AZN and RSGL 100 mg% were prepared in methanol. The solutions were maintained at − 4 °C for at least 4 days to ensure their stability.

### Construction of calibration curves

#### For spectrophotometric measurement of AZN

In a 10-mL volumetric flask, an exact volume of 0.1 mL of CDs stock solution was transferred. Deionized water was used to make the volume to mark, resulting in a final concentration of 0.5 μg/mL. This solution was scanned. A volume of 0.1 mL of CDs standard stock solution was transferred into a series of 10-mL flasks separately. To get the concentration range shown in Table 1, serial aliquots of standard AZN solution were added. Flasks were mixed well and completed to mark with deionized water. Absorbance difference (Δ*A*) for each concentration was measured at 238 nm (*λ*max). Linearity was constructed between calculated Δ*A* and the corresponding AZN concentrations.

**Table 1:** Assay parameters for the determination of AZN and RSGL by the proposed methods

Parameters	Spectrophotometric method	Fluorimetric method			
	AZN	*F*_o_ − *F* AZN	*F*_o_/*F* AZN	*F*_o_ − *F* RSGL	*F*_o_/*F* RSGL
*λ*_*nm*_	238	*λ*em 528	*λ*em 528	*λ*em 528	*λ*em 528
Conc. range (μg/mL)	5–30	0.001–0.005	0.001–0.005	0.001–0.005	0.001–0.005
Regression equation					
*a*^a^	-0.0364	15.298	1.041	31.59	0.755
*b*^b^	0.012	5178	16.68	81.95	4.805
*r*^c^	0.9996	0.9995	0.9998	0.9997	0.9997
*S*_*a*_	0.00338	0.302	0.4507	0.654	0.036
*S*_*b*_	0.00017	91.14	0.00149	1.979	0.108
*S*_*b*_^2^	2.89 × 10^−8^	8306.49	2.22 × 10^−6^	3.916	0.0118
*S*_*b*_ %	1.491	0.0058	0.0089	0.798	0.749
LOD	0.928	0.00019	0.0002	0.026	0.0247
LOQ	2.81	0.00058	0.0008	0.079	0.0749
Significance *F*	0.00022235	1.2	4.33	0.0153	0.0144
*F*	4495.82	3227.21	1369.82	1714.1	1948.43
Accuracy (mean ± RSD%)	99.60 ± 0.82	100.29 ± 0.18	99.72 ± 0.33	99.84 ± 0.42	101.03 ± 0.44
Precision (RSD%)					
Intraday precision	1.07	0.17	0.40	0.33	0.49
Inter day precision	0.58	0.19	0.41	0.51	0.36

^a^*a* is the intercept

^b^*b* is the slope

^c^*r* is the regression coefficient

#### For fluorimetric measurement of AZN and RSGL

By micropippetting 0.01 mL, from standard stock CDs solution into 10-mL volumetric flask the dilution step was made. To make the final concentration of 0.05 μg/mL, deionized water was added to the mark. Emission fluorescence of the prepared solution was measured at *λ*_em_ 528 nm, after its excitation at *λ*_ex_ 376 nm. Similarly, 0.01 mL of standard stock CDs solution was quantitatively transferred into two 10-mL flask sets. Serial aliquots of AZN and RSGL were added to each set separately and completed to marks with deionized water. Final concentration ranges for F measurements of both drugs were as stated in Table 1. The CDs solution (*F*_o_) and each solution of both drugs with CDs (*F*) were investigated for relative fluorescence intensities (*λ*_ex_ = 376 nm, *λ*_em_ = 528 nm). Calibration curves; Δ*F* (*F*_o_ − *F*) and *F* ratio (*F*_o_/*F*) were calculated and plotted against the concentrations of each drug separately.

### Preparation of pharmaceutical formulations

#### XITHRONE® 200 mg/5 mL powder for oral suspension

Powder was reconstituted with provided water into white homogenous suspension. Gentle mixing was made prior to sample pipetting. Portion of suspension, equal to 10 mg AZN, was pipetted into a 10-mL volumetric flask. Methanol was added for dissolution and flask was made up to mark (sample stock solution of 1 mg/mL).

#### DELZOSIN® 500 mg tablets

Five tablets were finely powdered after being exactly weighed. A 50-mL volumetric flask was filled with a portion of the tablet powder equivalent to 50 mg AZN. For drug extraction, a volume of 35 mL methanol was added to the powder and the flask was sonicated for 30 min. The sample solution was diluted to mark and filtered using 0.45-μm millipore membrane filter.

#### PARKINTREAT® 1 mg tablets

Fifteen Parkintreat® tablets were weighed and finely powdered in a specific manner. Methanol was added to an amount of powder proportional to the average weight of one tablet in a 10-mL volumetric flask. The flask was sonicated for 30 min and diluted to volume with methanol. A 0.45-μm millipore membrane filter was used to filter the sample solution.

## Results

Luminescent CDs were synthesized from garlic peels through a green and eco-friendly manner [10, 11]. They were fully characterized as illustrated in Additional file 1 and utilized as sensing probe to detect and quantify two nitrogenous non-fluorophoric drugs; azalide antibiotic, AZN and anti-parkinsonism, RSGL. In the present study, CDs’ solution is prepared in deionized water; to prevent any possible interferences in distilled water.

### Characterization of synthesized CDs

Carbon dots were synthesized and all characterization techniques in addition to quantum yield were done and previously reported [10].

### Analytical validation

The methods were validated following ICH guidelines [30].

#### Linearity

Calibration curves were constructed as previously mentioned. Table 1 indicates the regression and statistical parameters. Good correlation coefficient values with small intercepts were obtained. The detection limits (DL) and quantitation limits (QL) were calculated regarding the ICH guidelines equations.

#### Accuracy and precision

According to linearity ranges, three concentrations of selected drugs were analyzed three times (*n* = 3). This method was investigated in order to determine the drug's recovery at different levels. The more significant recoveries and low percentage error, the high accuracy were indicated, Table 1.

### Analysis of pharmaceutical formulations

Direct determination of AZN suspension and tablets, as well as RSGL in tablet formulation, were carried out using the proposed spectrometric methods. The analysis revealed that the recovery% and RSD% values were satisfactory Table 2. The Student's *t*-test and the variance ratio *F*-test were used to compare the results to a previously reported spectrophotometric method [31, 32]. The obtained *t*- and *F*-values did not reach the threshold levels Table 2, indicating a high level of agreement amongst the proposed and reported methods.

**Table 2:** Application of the proposed methods for determination of AZN and RSGL for five determinations

Pharmaceutical preparation	Mean% recovery ± SD^a^		Reference method [31]
	Spectrophotometric method	Fluorimetric method	
*AZN*			
XITHRONE® Suspension	98.78 ± 0.72	99.78 ± 0.36	100.49 ± 1.45
*t*^b^	2.62	1.60	
*F*^b^	5.05	4.96	
DELZOSIN® 500 mg tablets	99.77 ± 0.58	100.14 ± 0.93	
*t*^b^	2.70	2.23	
*F*^b^	3.97	5.16	

Pharmaceutical preparation	Fluorimetric method	Reference method [32]
*RSGL*		
PARKINTREAT® 1 mg Tablets	100.2 ± 0.75	100.45 ± 1.24
*t*^b^	0.41	
*F*^b^	5.39	

^a^Mean ± standard deviation of three determinations

^b^COMPARATIVE method represents the tabulated data of *t* and *F* at *P* = 0.05

## Discussion

### UV-spectrophotometric measurements

**Fig. 2:**
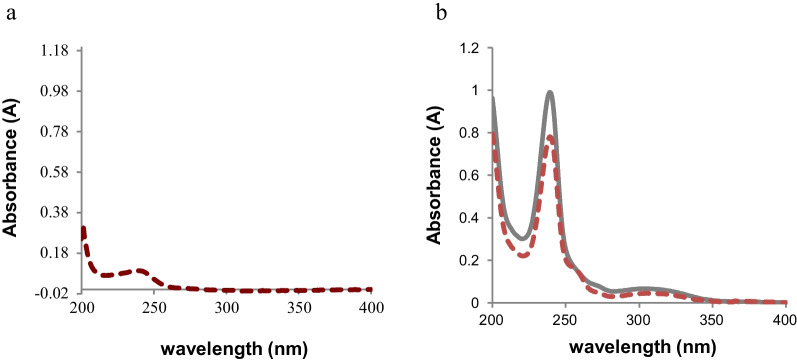
**a** Absorption spectra of 10 μg/mL AZN in methanol and **b** Absorption spectra of 50 μg/mL CDs and 20 μg/mL AZN after interaction with CDs in deionized water

Aqueous solution of CDs, 50 μg/mL, exhibits a pronounced absorption peak *λ*max at 238 nm. Standard AZN 10 μg/mL exhibits weak absorption spectrum as its structure lacks a chromophoric functional group. The chemical reaction between AZN and CDs is observed by the decrease in the absorbance value of CDs, measured at its *λ*max 238 nm, Fig. 2.

### Fluorimetric measurements

**Fig. 3:**
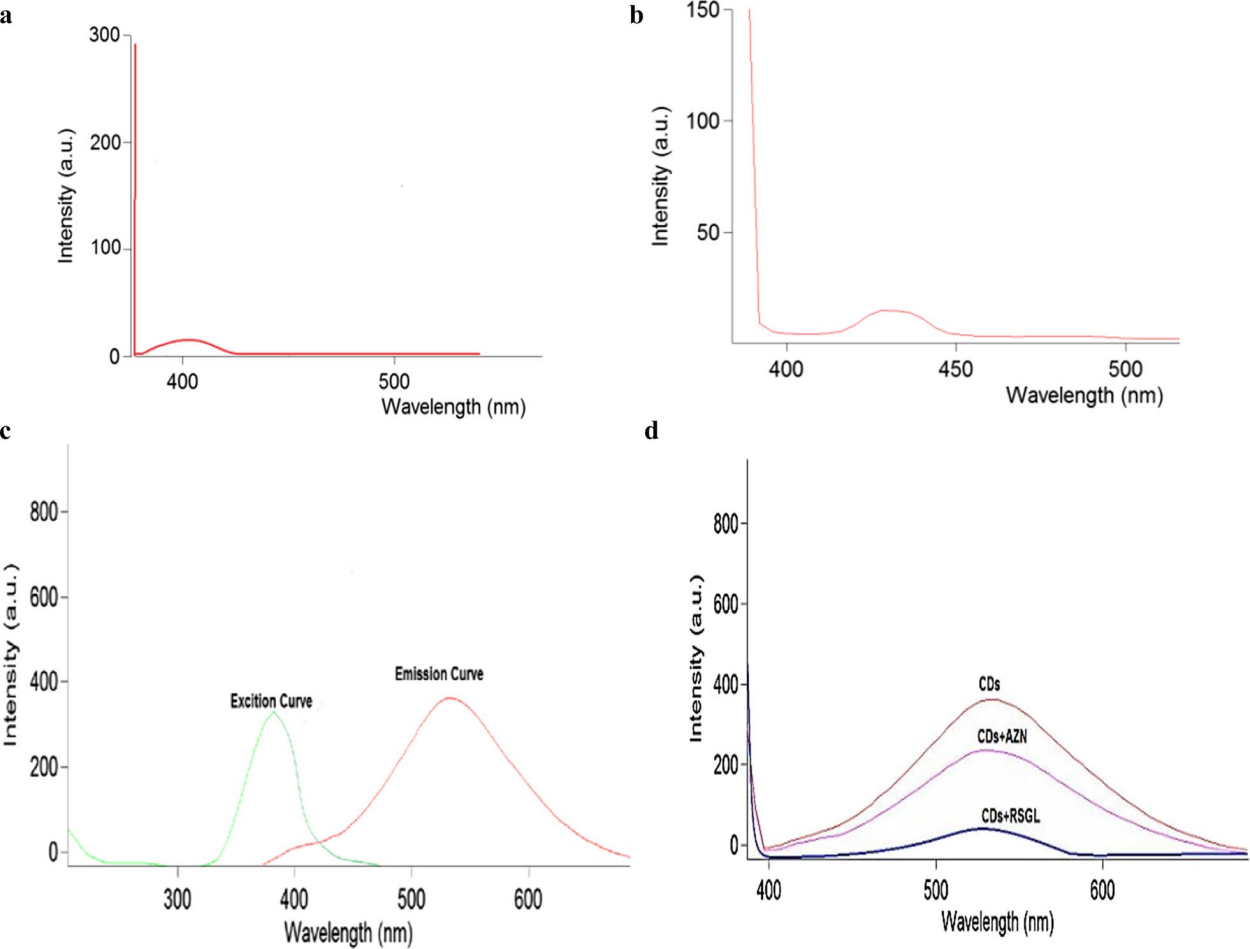
Fluorescence emission of **a** 0.1 μg/mL AZN, **b** 5 μg/mL RSGL, **c** Overlay for excitation and emission curves of CDs and **d** Quenching of CDs fluorescence emission in the presence of 0.001 μg/mL AZN and 0.25 μg/mL RSGL

Similarly, aqueous CDs solution exhibits intrinsic potent fluorimetric emission at *λ*em 528 nm, upon excitation at *λ*_ex_ 376 nm. Significant decrease in fluorimetric emission of CDs solution occurs upon adding traces of AZN and RSGL standard solutions, Fig. 3.

Nano CDs’ active surfaces undergo stoichiometric reactions with AZN and RSGL; owing to the presence of the two tertiary amine functional groups in AZN structure, and secondary amine in RSGL structure [28, 29] (Fig. 1). AZN traces can be directly quantified by the luminescence diminish of the nanodots. Absorbance difference Δ*A* (*A*_o_ − *A*), is linearly related to AZN concentrations. Where *A*_o_ is the absorbance value of CDs alone and *A* is the absorbance value of CDs with standard AZN solution, both measured at 238 nm. Furthermore, CDs solution fluorescence at 528 nm, upon excitation at 376 nm. Each of AZN and RSGL traces interact with dots and decrease the CDs’ emission. Drugs’ concentrations are efficiently calculated using Stern–Volmer Equation (Eq. 1). Decrease in fluorescence (Δ*F* = *F*_o_ − *F*) is linearly correlated to molar concentration of quencher (AZN or RSGL). Where *F*_o_ is the relative fluorescence intensity of CDs alone and F is relative fluorescence intensity of CDs with standard AZN solution.

1 $$\Delta F\left( {F_{o} {-}F} \right) = 1 + K\left[ Q \right]$$

i.e. fluorescence intensity difference as a function of the quencher concentration [*Q*] is linear with intercept 1 and slope *K*.

The effect of diluting solvent was studied by trying different solvents such as acetonitrile, acetone, methanol and water. An aliquot of the stock solution of CDs was diluted with different diluting solvents to obtain a concentration of 0.05 μg/mL, and the emission intensity was recorded. Both water and methanol gave highest intensity of emission. But water was selected to obtain green analytical method.

The influence of pH value on quenching effect of AZN or RSGL on CDs was also studied. It was found that different pH values did not have any effect on Δ*F*. So, to increase simplicity of method water was used not buffer solution.

## Conclusions

In summary, Carbon nanodots that are prepared from garlic peels showed an excellent emission fluorescence peak at 528 nm after excitation at 376 nm. For the first time, CDs are utilized as spectrometric probes for the assay of two non-fluorophoric nitrogenous pharmaceutical entities in bulk and formulated tablets. AZN traces decrease the absorption and fluorimetric emission of CDs in a linear correlation. Alternatively, RSGL quenches the CDs’ fluorimetric emission linearly. Furthermore, this sensor offers several positive traits, including simplicity of synthesis, ecofriendly water usage in sample preparation, a quick response and remarkable selectivity. This recommends the applying of the proposed methods for the estimation of AZN and RSGL in green and economic conditions.

## Supplementary Information

**Additional file 1.** Synthesis and specific characterization of used CDs.

## Data Availability

All data generated or analyzed during this study are included in this published article [and its supplementary information files].

## Abbreviations

CDs: Carbon dots
AZN: Azithromycin
RSGL: Rasagiline
DL: Detection limits
QL: Quantitation limits
*λ*max: Maximum wavelength
RSD: Relative standard deviation
*Q*: Quencher concentration
HPLC: High-performance liquid chromatography
