# Supplementary material for: Carbon dots as fluorescent nanoprobes for assay of some non-fluorophoric nitrogenous compounds of high pharmaceutical interest
Source: Beni Suef Univ J Basic Appl Sci. 2023 Jan 17;12(1):8. doi: 10.1186/s43088-023-00346-z (PMC9844168; doi:10.1186/s43088-023-00346-z)
Supplement: Supplementary file 1 — Additional file 1. Synthesis and specific characterization of used CDs. [file 43088_2023_346_MOESM1_ESM.docx]

**Additional file 1**

Carbon dots were prepared from garlic peels at 250 ^o^C in atmospheric oxygen. Characterization techniques proved that the prepared carbon dots with a particle size around of 3.2 nm, amorphous in nature, and rich with different C-O functional groups.

**Preparation of carbon dots**

Carbon nanodots (C-dots) were synthetized by a method as described by Gaber et al [1] with slight modifications. Garlic peels were cleaned with deionized water and dried at 60 ^o^C for 3 hours. The dried material was grinding into pieces of about 0.5 mm. 10 grams of the ground dried materials were added into a cleaned dried crucible and heated at 250 ^o^C for 2 hours in atmospheric oxygen. The residue was then dissolved in 20 mL Milli-Q water, filtered through 0.45 μm nylon filter. The filtrate was purified via dialyzer tube for two days using MWCO, 3.5 KDa.

**Characterization of carbon dots**

Carbon dots were investigated using different techniques. Shimadzu DTA-50, Japan used for the investigation of thermogravimetric analysis curve (TGA) of carbon nanodots from room temperature to 900 ^o^C. X-ray diffraction pattern (XRD) was applied by using PANalytical X’Pert PRO, Cu Kα radiation (λ=1.5405 A°) in 2θ range of 5‒80 degree where the tube was operated at 30 kV. Transmission electron microscopy (TEM) and energy dispersive X-ray spectra (EDX) were studied at an acceleration voltage of 200 kV by using a JEOL‒JEM-2100 (Tokyo, Japan). Fourier transforms infrared spectroscopy (FTIR) was applied by using a Mattson 5000 FTIR spectrometer in the range 400‒ 4000 cm^-1^.

least one week with keeping in the refrigerator. It was found that re-analyzing data of the sample proves that there is no indication of any change of carbon dots.

**Characterization of carbon dots and factors affecting its fluorescence**

TGA of C-dots is displayed in Fig.2A. Weight loss at a temperature between 130 and 200 ^o^C (2.5‒4%) is related to the adsorbed water molecules and weakly attached species through weak hydrogen bonds [2]. Weight loss at about 500 ^o^C (~5.5%) is related to the surface ‒OH functional groups decomposition. Sharp weight loss (12‒91%) in the range 685‒805 ^o^C can be related to oxidation of the C-dots indicating the thermal instability of C-dots at higher temperatures [3]. X-ray diffraction patterns for C-dots are displayed in Fig.2B. The reflection band at 2θ= 21.68° (002) and the broad band at 2θ= 43.1° {100/101} are related to the diffraction of graphite crystal planes [4]. The average particle size was determined for (002) band using Debye-Scherrer's equation (Eq. 1).

 (1)

Where, *λ* (X-ray wavelength) = 1.5405 Å, *k* equals 0.9, and *β* is the full width at half peak height. *P_s_* was calculated to be 7.5 Å. Bragg’s equation was applied to calculate the interlayer spacing and was found to be 4.34 Å. Fig. 2C depicts TEM image of C-dots which showed semispherical and smaller nanoparticles with an average diameter of 3.2 nm as indicated in the histogram (Fig.2D). Based on the histogram (Fig.2D) which was constructed based on 100 particles in different micrograph regions, we found that about 60% of C-dots particles located between 2 and 4 nm particle size and the same result was observed by De and Karak [5]. Selected area electron diffraction (SAED) patterns of the sample (S1.A) revealed a diffused ring confirming the amorphous nature of the prepared C-dots [6, 7]. EDX analysis of C-dots sample (S1.B) shows that surface carbon contents about 95.4% while oxygen represents about 4.5% of the total surface atoms.

1. H. Gaber, B. Rosana, A. Josefa and G. Marta, Beilstein Journal of Nanotechnology, **7** 758–766(2016).
2. M. Ashmi, P. Sunil, Th. Mukeshchand, J. Dhanashree and Sh. Madhuri, Journal of Materials Chemistry B., **2,** 698–705 (2014).
3. O. Emil, A. Zhypargul, I. Chihiro, I. Hirotaka, U. Saadat and M. Tsutomu, Journal of Nanoscience and Nanotechnology, **15**, 3703–3709 (2015).
4. F. Suárez-Garcı́a, A. Martínez-Alonso and J. M. Díez Tascón, Journal of Analytical and Applied Pyrolysis, **63,** 283–301 (2002).
5. B. De and N. Karak, RSC Adv., **3**, 8286 (2013).
6. P. Tathagata, M. Shanid and P. Gopinath, ACS Omega, **3**, 831–843(2018).
7. J. Liang, J. Wanga, K. Yu, K. Song, X. Wang, W. Liu, J. Hou and C.Liang, Chemical Physics, **528**, 110538–110544(2020).


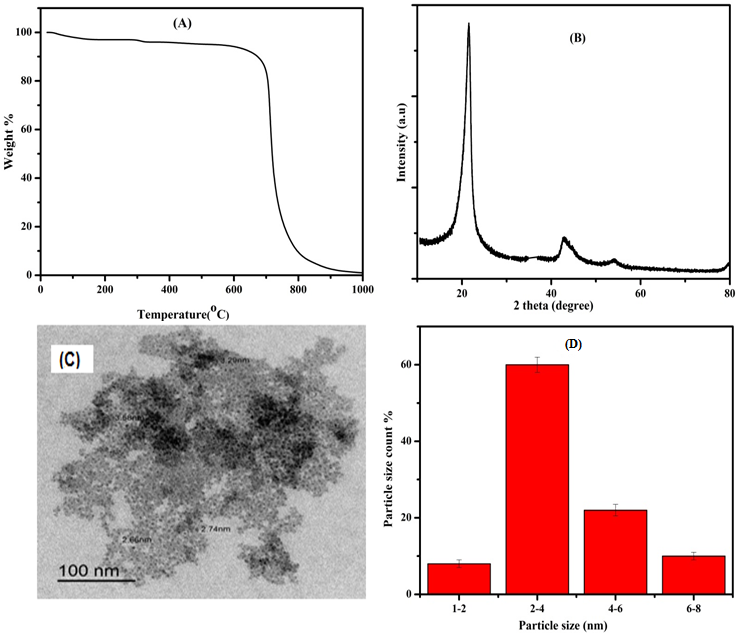


**Fig. 1.** Thermogravimetric analysis curve (A), X- ray diffraction pattern (B), transmission electron microscopy (C) and histogram (D) for the synthetized carbon nanodots.
